# Genomic Features and Phylogenetic Analysis of Antimicrobial-Resistant *Salmonella* Mbandaka ST413 Strains

**DOI:** 10.3390/microorganisms12020312

**Published:** 2024-02-01

**Authors:** Valdinete P. Benevides, Mauro M. S. Saraiva, Camila F. Nascimento, Enrique J. Delgado-Suárez, Celso J. B. Oliveira, Saura R. Silva, Vitor F. O. Miranda, Henrik Christensen, John E. Olsen, Angelo Berchieri Junior

**Affiliations:** 1 Postgraduate Program in Agricultural Microbiology, School of Agricultural and Veterinarian Sciences, São Paulo State University (Unesp), Jaboticabal 14884-900, Brazil; valpbenevides@gmail.com; 2 Department of Pathology, Reproduction and One Health, School of Agricultural and Veterinarian Sciences, São Paulo State University (Unesp), Jaboticabal 14884-900, Brazil; 3 Department of Veterinary and Animal Sciences, University of Copenhagen, 1870 Frederiksberg, Denmark; 4 Facultad de Medicina Veterinaria y Zootecnia, Universidad Nacional Autónoma de México (UNAM), Mexico City 04510, Mexico; 5 Center for Agricultural Sciences, Department of Animal Science, Federal University of Paraiba (CCA/UFPB), Areia 58051-900, Brazil; 6 Global One Health Initiative (GOHi), The Ohio State University, Columbus, OH 43210, USA; 7 Laboratory of Plant Systematics, Department of Biology, School of Agricultural and Veterinarian Sciences, São Paulo State University (Unesp), Jaboticabal 14884-900, Brazil

**Keywords:** antimicrobial resistance, bacteriophage, One Health, pathogenesis, salmonellosis, virulence factors

## Abstract

In recent years, *Salmonella enterica* subsp. *enterica* serovar Mbandaka (*S.* Mbandaka) has been increasingly isolated from laying hens and shell eggs around the world. Moreover, this serovar has been identified as the causative agent of several salmonellosis outbreaks in humans. Surprisingly, little is known about the characteristics of this emerging serovar, and therefore, we investigated antimicrobial resistance, virulence, and prophage genes of six selected Brazilian strains of *Salmonella* Mbandaka using Whole Genome Sequencing (WGS). Multi-locus sequence typing revealed that the tested strains belong to Sequence Type 413 (ST413), which has been linked to recent multi-country salmonellosis outbreaks in Europe. A total of nine resistance genes were detected, and the most frequent ones were *aac(6′)-Iaa*, *sul1*, *qacE*, *bla*_OXA-129_, *tet(B)*, and *aadA1*. A point mutation in ParC at the 57th position (threonine → serine) associated with quinolone resistance was present in all investigated genomes. A 112,960 bp IncHI2A plasmid was mapped in 4/6 strains. This plasmid harboured tetracycline (*tet*ACDR) and mercury (*mer*) resistance genes, genes contributing to conjugative transfer, and genes involved in plasmid maintenance. Most strains (four/six) carried *Salmonella* genomic island 1 (SGI1). All *S.* Mbandaka genomes carried seven pathogenicity islands (SPIs) involved in intracellular survival and virulence: SPIs 1-5, 9, and C63PI. The virulence genes *csgC*, *fimY*, *tcfA*, *sscA*, (two/six), and *ssaS* (one/six) were absent in some of the genomes; conversely, *fimA*, *prgH*, and *mgtC* were present in all of them. Five *Salmonella* bacteriophage sequences (with homology to *Escherichia* phage phiV10, *Enterobacteria* phage Fels-2, *Enterobacteria* phage HK542, *Enterobacteria* phage ST64T, *Salmonella* phage SW9) were identified, with protein counts between 31 and 54, genome lengths of 24.7 bp and 47.7 bp, and average GC content of 51.25%. In the phylogenetic analysis, the genomes of strains isolated from poultry in Brazil clustered into well-supported clades with a heterogeneous distribution, primarily associated with strains isolated from humans and food. The phylogenetic relationship of Brazilian *S*. Mbandaka suggests the presence of strains with high epidemiological significance and the potential to be linked to foodborne outbreaks. Overall, our results show that isolated strains of *S.* Mbandaka are multidrug-resistant and encode a rather conserved virulence machinery, which is an epidemiological hallmark of *Salmonella* strains that have successfully disseminated both regionally and globally.

## 1. Introduction

Foodborne diseases caused by *Salmonella* spp. have been frequently associated with poultry products [1]. According to the European Food Safety Authority (EFSA), 65,208 cases of salmonellosis in humans were reported in 2022 [2]. More than 2.650 serovars of this pathogen have already been identified, of which about a hundred have been isolated from both animals and humans [3], wherein in humans, the serovars *S*. Enteritidis (54.6%), *S*. Typhimurium (12.1%), *S.* Typhimurium single-phase 1,4,[5],12:i:- (10.4%), and *S*. Infantis (2.3%) the main isolates [2]. 

The epidemiological importance of *Salmonella* serovars is related to their geographically widespread distribution and their ability to infect multiple hosts [4]. For instance, day-old chicks can arrive at poultry farms already infected with several *Salmonella* serovars [5]. Moreover, vectors including rodents play a central role in the maintenance and dissemination of *Salmonella* within poultry flocks [5].

*Salmonella* Mbandaka (*S*. Mbandaka) is a bacterial pathogen with diversity in its host range that includes bovines, poultry, and humans [2,6,7]. However, despite the identification of *S*. Mbandaka in other sources, including animal feed [8], the prediction of the Sequence Type (ST) is not always investigated in studies. Recently, the dissemination of *S*. Mbandaka ST413 in poultry farms has been reported [6] and in humans [7]. This clone was involved in a multi-country outbreak linked to the consumption of poultry meat in EU/EEA, Israel, and the UK, which resulted in 196 human cases, 19 hospitalizations, five septicaemic infections, and one death [7]. Likewise, this serovar was also related to multistate outbreaks in the United States [9]. Another emergent concern to public health is the multidrug-resistant (MDR) profiles exhibited by *S.* Mbandaka isolates from poultry farms in different countries, which involves tetracycline, fluoroquinolones, aminoglycosides, sulphonamides, and third-generation cephalosporins [10,11].

This combination of virulent and MDR phenotypes has been associated with invasive infections caused by *S*. Mbandaka, worsening the severity of clinical signs, and leading to higher mortality rates in humans [7,12]. Despite the emergent epidemiological importance of *S.* Mbandaka, there is little information on the genomic features that allow this serovar to persist in poultry production facilities and use poultry as asymptomatic carriers to disseminate. Thus, we carried out an in-depth genomic characterization of *S.* Mbandaka isolated from the caecal content of laying hens in São Paulo State, Brazil, to analyse the evolutionary, epidemiological, and adaptive potential of this serovar in poultry and humans.

## 2. Materials and Methods

### 2.1. Sample Collection

Six strains of *S*. Mbandaka were selected from a collection of 29 isolates using the antimicrobial resistance phenotypic profile observed in a previous study by our group as selection criteria, spanning the period between 2016 and 2017 [13]. In this study, a pool of caecal content samples was collected in 151 commercial layer farms located in the Midwest region of the São Paulo State, Brazil. In total, 2008 samples of 300 g each were collected in sterile flasks and refrigerated (4–8 °C) until analyses of isolation and identification as described previously [13]. The selected six strains were isolated between 2016 (1092/18, 1095/18, 1096/18, and 1097/18) and 2017 (1124/18 and 1158/18) from the caecal content from live laying hens and a laying quail (the isolate 1092/18) from commercial egg-laying farms.

### 2.2. Whole Genome Sequencing (WGS)

The genomic DNA of the strains was extracted using the Maxwell RSC Cultured Cells DNA kit (Promega, Madison, WI, USA). After extraction, DNA integrity was evaluated by electrophoresis in 1% agarose gel, quantified in a spectrophotometer (NanoDrop, Thermo Fisher Scientific, Waltham, MA, USA), and the concentration analyses using Qubit dsDNA HS Assay kit (Thermo Fisher Scientific, Scoresby, Australia). A DNA library was prepared using the Flex DNA Library Preparation Kit (Illumina, San Diego, CA, USA) following the instructions from the supplier, followed by paired-end sequencing performed in Illumina MiSeq (Anicon, Germany; NGS-MiSeq, University of Copenhagen, Denmark) using a Nextera XT v3 kit (2 × 300 bp insert size).

### 2.3. Downstream Bioinformatic Analyses

The quality of raw reads was analysed using FastQC 0.11.9 [14]. Low-quality reads and adapters were removed using Trimmomatic 0.39 [15], before assembling genomes using Unicycler [16]. The resulting assembled sequences were automatically annotated with RAST 2.0 [17]. Utilizing bioinformatics tools available from Center for Genomic Epidemiology (CGE), including SeqSero 1.2 [18], and MLST 2.1 [19,20], the assembled sequences underwent in silico analysis. This analysis aimed to confirm the serotype and determine the Multilocus Sequence Typing (MLST) sequence type (ST) for *Salmonella enterica*, respectively. 

The identification of *Salmonella* pathogenicity islands (SPIs) was performed using SPIFinder 2.0 [21] on April 6th, 2023. The results were compared by nucleotide alignment with the BLAST Ring Image Generator (BRIG) 0.95, in which *Salmonella* Typhimurium LT2 (NC_003197.2) was used as a reference strain [22]. Using Blast atlas analysis, conducted at the Gview server [23], we predicted the presence of SGI1 in the study genomes. For that purpose, we used the reference sequence of *Salmonella* Typhimurium genomic island I (AF261825.2) and ran the analysis with default BLAST parameters. This approach included only those query genome files that were found to harbour SGI1, as indicated by prior results generated by SPIFinder. The output file obtained was visualized using GView’s tools, facilitating the investigation of both conserved and variable regions [23].

We also conducted a detailed analysis of the SPI 2-3 regions of *S.* Mbandaka. This investigation was prompted by observed variations within these islands, to elucidate the genetic nuances and potential associated functional implications. To accomplish this, sequences of SPI 2-3 regions from the six *S.* Mbandaka strains were initially collected, alongside the reference sequence of *Salmonella enterica* subsp. *enterica* serovar Typhimurium LT2 (NC_003197.2). Base-to-base alignment was performed using Seaview 5.0.4 [24], and posteriorly, multiple sequence alignment was carried out using the Clustal Omega tool within Seaview software to reveal the genetic variations and similarities. The aligned regions were curated using Geneious [25].

The VFanalyzer tool available in the Virulence Factors Database [26] was utilized to detect putative virulence factors in the investigated *Salmonella* genomes, and *Salmonella* Typhimurium LT2 (chromosome: NC_003197) was used as the reference genome; this analysis was conducted on 6 April 2023. These data on the presence or absence of virulence determinants were used to generate a heatmap using Morpheus (https://software.broadinstitute.org/morpheus, accessed on 24 August 2023). Additionally, the CGE web server was used on 5 April 2023 to identify the presence of antimicrobial resistance genes using ResFinder 4.1 [27], whereas the detection of plasmid replicons was conducted with PlasmidFinder 2.0 [28] and manually curated using Geneious [25]. For this last analysis, the *S*. Mbandaka 1095/18 genome was aligned with the R478 plasmid (BX664015) using the Map to Reference tool with the default setting to build the contig [25]. Incongruent sequences were removed before circularizing the mobile genome.

The six assembled genomes were analysed by Phage Search Tool Enhanced Release (PHASTER) to identify the presence of prophages [29,30]. Only prophages identified as “intact” (score > 90) were considered for the results. The genome alignment tool [31] was used to assess linkages and correlations based on nucleotide identity between phage sequences.

To elucidate phylogenetic relationships amongst *S*. Mbandaka isolates, whole-genome phylogenies were reconstructed using *Escherichia coli* (U00096.2), *Shigella flexneri* (AE014073.1), and *Salmonella* Typhimurium LT2 (AE006468.2) as outgroups. In addition to the six genomes that were sequenced as part of this research, 475 assembled sequences of *S*. Mbandaka were gathered from the Enterobase database [32,33], spanning various isolation sources over the past five years (2019 to 2023). The genomes were selected on 15 August 2023, in the database according to the standards established by the authors, using the following filters: *S.* Mbandaka, ST413, and coverage ≥ 90. Moreover, the genomes were recovered already assembled (files in “.fasta” format). Further details of each of the isolates are available in the Supplementary Materials (Table S1). Input genomes were annotated with Prokka 1.14.5 [34] using default parameters. The Roary v3.12.0 [35] pipeline with default parameters generated the matrix from the MAFFT alignment program [36]. The phylogenetic tree was constructed using the Maximum Likelihood method in IQ-TREE2 2.2.2.6 [37] with clade support estimates calculated using ultrafast bootstrap (UFBoot) of 1000 pseudoreplicates [38]. TN+F+R10 was applied as a best-of-fit model according to the BIC with ModelFinder [39]. To edit the phylogenetic tree, the online tool iTOL—Interactive Tree of Life was used [40].

### 2.4. Data Availability and Accession Numbers

The assembled genomes of six *Salmonella enterica* subsp. *enterica* serovar Mbandaka strains were deposited at the NCBI Sequence Read Archive (SRA) website, under Project PRJNA1015686. The complete genome data of 1092/18, 1095/18, 1096/18, 1097/18, 1124/18, and 1158/18 were deposited in GenBank with Accession No. SAMN37152295, SAMN37152294, SAMN37152296, SAMN37152297, SAMN37152298, and SAMN37152299, respectively.

## 3. Results

### 3.1. Genome Assembly, Genome Annotation, and MLST

Characteristics and quality parameters of the draft genome assemblies are shown in Table 1. Genomes had GC content between 51.8 and 52.2% and genome sizes between 4,738,760 and 4,934,713 bp, which are within the typical range of *S. enterica* (*Salmonella* Typhimurium LT2, NC_003197.2). MLST analysis showed that all strains belonged to the sequence type 413 (ST413).

### 3.2. Antimicrobial Resistance Determinants

All isolates harboured the gene *aac(6′)-Iaa*, which encodes resistance to amikacin and tobramycin, while the gene *aadA1*, responsible for conferring resistance to spectinomycin, was found in four out of six strains. The genes *sul1* (sulfonamides), *dfrA21*, and *dfrA25* (trimethoprim) were also found in five/six, four/six, and one/six of strains, respectively. *tetA* (one/six) and *tetB* (four/six) encoding tetracycline resistance encoding genes were detected in some strains, while the *bla*_OXA-129_ gene related to resistance to β-lactams was detected in four/six of the genomes. Additionally, our results also disclosed the widespread distribution of the *qacE* gene (five/six) in these *S.* Mbandaka strains, which contributes to the resistance to quaternary ammonium compounds (QAC). Moreover, point mutation in ParC [T57S], which confers resistance to quinolones, was identified in all the strains (Table 2).

### 3.3. Plasmid Replicons and Homology to Published Plasmid Sequences

Most strains (five/six) of *S.* Mbandaka harboured plasmid replicons, except the strain 1097/18. IncHI2A plasmid replicons were detected in four genomes (1095/18, 1096/18, 1124/18, and 1158/18), whereas a replicon of IncN type was identified in a single strain (1092/18) (Table 3). The detection of those replicons identified in known plasmids (R478 and R46) suggested the presence of a similar sequence to these plasmids in the genomes under study, which was further confirmed by aligning the reference sequence of plasmid R478 (BX664015) to genome 1095/18. The p109518 plasmid was assembled using R478 as a reference genome aligning this to the sequence of 1095/18. While the IncN replicon plasmid (R46 homologous plasmid) was predicted in the 1092/18 genome, attempts to circularize it were unsuccessful. Only a limited region of 6823 bp was identified, containing the *ccgEIII*, *ardR*, *ardB*, *mucA*, *mucB*, *mpr*, *ardK*, and *repA* genes.

The results are based on raw reads with an identity threshold of 95% and a minimum coverage of 60% [28]. 

**Figure 1:**
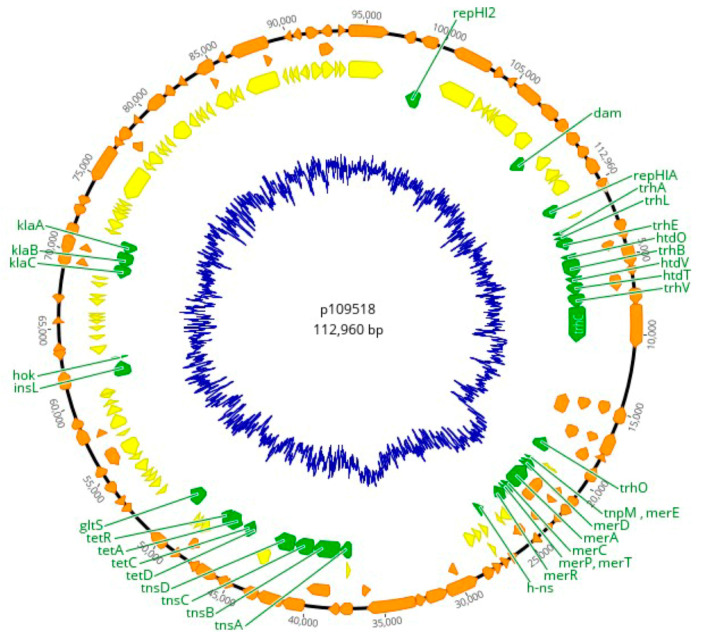
The predicted structure of the 112,960 bp p109518 plasmid from *S.* Mbandaka (isolate 1095/18) shows backbone and accessory module regions. Genes with a predicted function are denoted by green arrows, hypothetical proteins in yellow, and ORFs in orange. The innermost blue circle presents a GC content of 44.3%.

The manual assembly of the putative R478-like plasmid generated a high-quality circular sequence with only one contig of 112,960 bp (named p109518) (Figure 1). The annotation of p109518 predicted 103 Open Reading Frames (ORFs), 79 Coding Sequences (CDSs), and 44.3% G+C content. The p109518 plasmid harbours 36 genes of known function, of which two are involved in replication initiation (rep), that is, genes that encode the core IncHI2 plasmid determinants such as repHIA, position 1…875 (length: 875) and repHI2, position 8463…99551 (length: 1089). The plasmid contained nine genes predicted to be involved in conjugation, including six *trh* genes (ABELOV) and three *htd* (OTV) genes. The *dam* gene encoding for a DNA adenine methylase was also detected. Other genes associated with adaptive advantages for the bacteria included *tet*-genes (ACDR), which encode tetracycline resistance. A genetic region related to mercury resistance was also detected, including the *mer* operon (ACDEPT). 

### 3.4. Analysis of SPIs and Virulence Determinants

**Figure 2:**
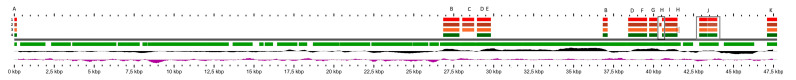
Linear BLAST atlas of SGI1 in *S.* Mbandaka strains 1158/18 (1), 1096/18 (2), 1095/18 (3), and 1092/18 (4). The backbone is represented in grey, the reference sequence (AF261825.2) is represented in a light shade of green, GC content is depicted in black, and GC skew is displayed in purple. A: *thdf* (product: tRNA-5-carboxymethylaminomethyl-2-thiouridine (34) synthesis protein MnmE), B: *intI1* (product: integron integrase Inti1), C: *aadA2* (product: aminoglycoside 3″-nucleotidyltransferase), D: *qacEdelta1* (product: small multidrug resistance (SMR) efflux transporter => QacE delta 1, quaternary ammonium compounds), E: *sul1delta* (product: dihydropteroate synthase type-2—sulphonamide resistance protein), F: *sul1* (product: dihydropteroate synthase type-2—sulphonamide resistance protein), G: product: similar to puromycin N-acetyltransferase, H: product: hypothetical protein, I: *tnpA* (product: transposase), J: *int2* (product: phage integrase) K: *yidY* (product: multidrug efflux pump MdtL (of MFS type)). The figure was built using the Blast Atlas tool (Gview server).

**Figure 3:**
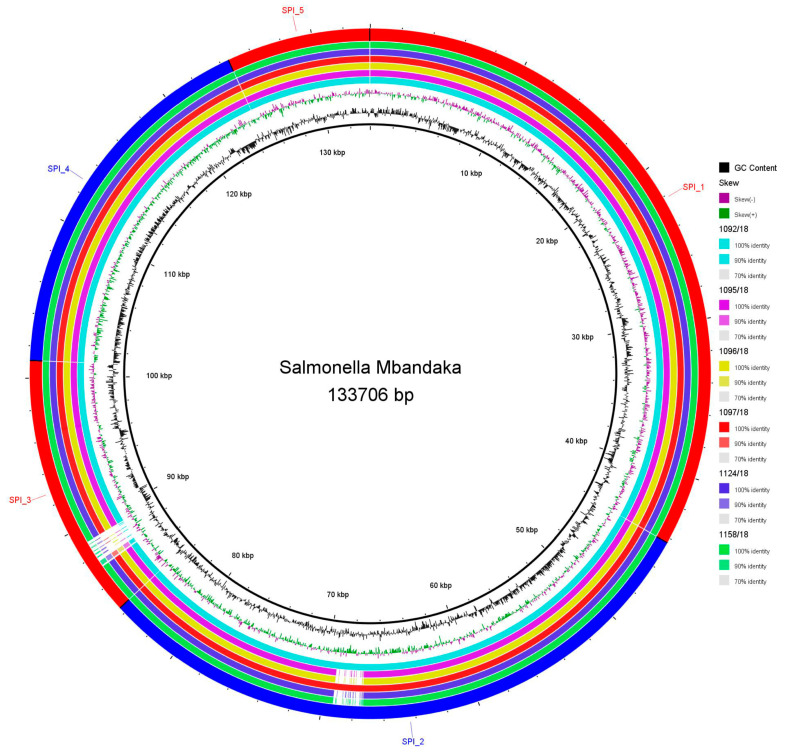
BLAST ring image of the SPIs detected in six *S.* Mbandaka isolates from laying hens and quail in São Paulo State, Brazil. Colour intensities represent the percentage of identity (>90%) with the reference strain *Salmonella* Typhimurium LT2, while blank areas indicate no identity with the reference. The figure is shown in order from inside to outside, starting from the isolates in the right column.

All the sequenced isolates carried seven SPIs: C63PI, SPI 1 to 5, and SPI 9. We also detected the presence of SGI1 containing an antibiotic-resistance gene cluster in 1095/18, 1092/18 1096/18, and 1158/18 sequences (Figure 2). After the local alignment of SPIs 1-5, a deletion close to the 5′ region of SPI 3 (~2000 bp) was the main difference observed between the study isolates and the reference *Salmonella* Typhimurium LT2 strain. The SPI 2 region, before 70 kbp in Figure 3, was shown to have variations in four of the *S.* Mbandaka genomes (except in genomes 1092/18 and 1097/18) when compared with the reference genome. Conversely, we found conserved full SPIs 1, 4, and 5 in all the sequenced isolates (Figure 3).

**Figure 4:**
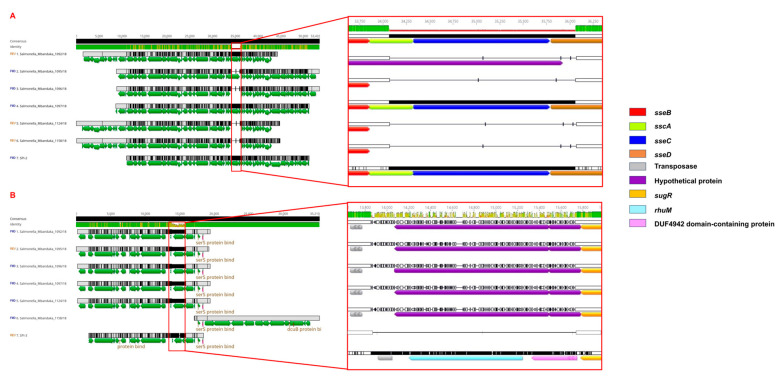
Comparative genomic analysis of SPIs 2 and 3. (**A**) Alignment of the SPI 2 locus and (**B**) alignment of the SPI 3 locus in *Salmonella enterica* serovar Typhimurium LT2 to six *S.* Mbandaka sequences (1092/18, 1095/18, 1096/18, 1097/18, 1124/18, and 1158/18).

Variations related to deletions within SPI 2 were detected in some genomes (1096/18, 1124/18, and 1158/18); in the 1095/18 genome, the variation occurred in a region encoding a hypothetical protein of unknown function (Figure 4). It is worth mentioning that the 1092/18 and 1097/18 genomes were identical to those found in the reference sequence. In SPI 3, the *rhuM* gene and the DUF4942 domain-containing protein were replaced by hypothetical proteins of 726 bp and 207 bp, respectively (except for genome 1158) (Figure 4).

The genomes under study carried multiple virulence genes, which were distributed into eight virulence factor (VF) classes. These included fimbrial and nonfimbrial adherence determinants, macrophage inducible genes, magnesium uptake, secretion systems, stress adaptation, and other adherence determinants. Some sequences of *S.* Mbandaka showed variations in relation to the presence of the fimbrial adherence determinants. For instance, the genes *csgC* (1092/18, 1097/18, 1124/18, and 1158/18), *fimY* (1095/18, 1092/18, 1097/18, and 1158/18), *tcfA* (1095/18, 1092/18, 1097/18, and 1124/18) and *stcB* (1095/18) were absent in some of the studied genomes (Figure S1). Conversely, genes involved in the macrophage inducible system (*mig-14*), magnesium uptake (*mgtCB*), and a two-component regulatory system (*phoPQ*) were detected in all the analysed genomes. Furthermore, the analysis highlighted the absence of *sseA*, a gene associated with the secretion system class in the *Salmonella* genus.

Several genes encoded in SPI-2’s type three secretion system (T3SS) were detected in all sequences. However, some absences were also observed for the *ssaI*, *ssaM*, *ssaS*, and *sscA* genes. The *sodCI* gene, which is involved in stress adaptation, was predicted in all genomes of *S.* Mbandaka. Likewise, the *fae* (CDEHI), *ste* (ABCDEF), and *stk* (ABCDEFG) operons related to bacterial adherence and fimbria were detected in all analysed genomes. Furthermore, three genes that are implicated in nonfimbrial adherence in *Salmonella* serotypes (*shdA* = 3/6; *ratB* = 5/6; *misL* = 6/6) were detected (Figure S1).

### 3.5. Prophage Regions in Genomes of S. Mbandaka

Overall, ten prophage sequences were identified in the genomes. The *Salmonella* phage SW9 prophage was uniquely detected in four (1095/18, 1096/18, 1124/18, and 1158/18) *S.* Mbandaka sequences. In contrast, the highest number of prophage sequences was detected in two other strains, which contained three prophage sequences per genome, including prevalent prophages such as *Escherichia* phage phiV10 (1092/18 and 1097/18), *Enterobacteria* phage Fels-2 (1092/18 and 1097/18), *Enterobacteria* phage HK542 (1092/18), and *Enterobacteria* phage ST64T (1097/18). We identified an extensive variation in genome phage sizes from *S.* Mbandaka sequences (24.7–47.4 kb) and % GC content (48.57–52.83%). More details about the predicted phages in *S.* Mbandaka genomes are described in Table 4. The genes found in all phage sequences involve proteins to prevent the degradation of viral genetic material, DNA packaging, phage structural proteins, lysis components, DNA recombination, regulation, and replication. No phage-borne virulence or antimicrobial-resistance genes were predicted in any of the six *S.* Mbandaka sequences (Figure S2).

### 3.6. Phylogenetic Insights into S. Mbandaka

**Figure 5:**
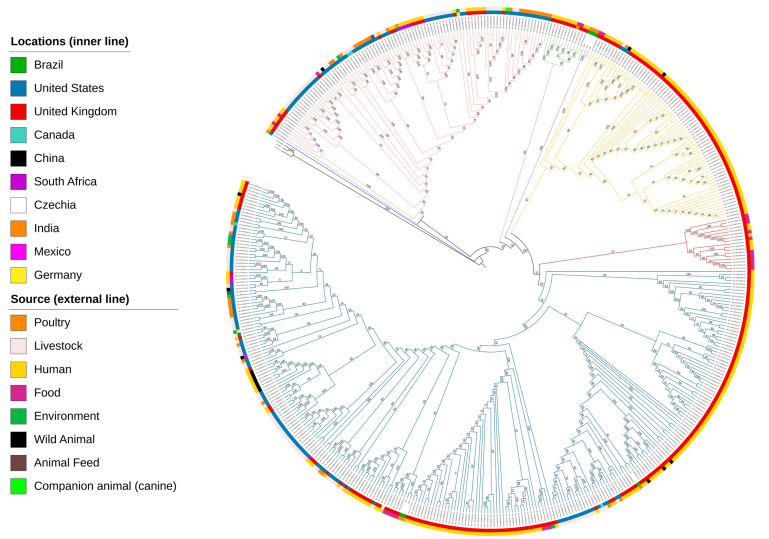
Maximum Likelihood phylogenetic tree of 481 ST413 *S.* Mbandaka strains recovered from poultry. *Escherichia coli* (U00096.2), *Shigella flexneri* (AE014073.1), and *Salmonella* Typhimurium LT2 (AE006468.2) were used as outgroups and to root the tree. The clade support is indicated above or next to each branch as bootstrap values, as calculated from 1000 pseudoreplicates. The colours in the inner ring of the tree represent the location of isolation and the colours in the outer ring indicate the source of isolation (see legend). The coloured branches represent the division of clades: clade 1 (light pink branch), clade 2 (green branch), clade 3 (purple branch), clade 4 (yellow branch), clade 5 (red branch), and clade 6 (blue branch).

The pangenome analysis indicated that all the analysed *S.* Mbandaka ST413 genomes (*n* = 481) harboured a total set of 16,663 genes, consisting of 3983 core genes, 203 soft-core genes, 513 shell genes, and 11,964 cloud genes. The 3983 core and soft-core genes, which represent a conserved set of genes shared among the majority of all the analysed genomes, were utilized as the basis for conducting the phylogenetic analysis. The Maximum Likelihood tree (Figure 5) revealed six clades, with two well-defined clades (purple and red) that distinctly grouped the genomes according to their geographical origin. 

The genomes SAL MC9044AA AS (United States—Poultry—2020) and SAL XC7954AA AS (United Kingdom—Human—2022) did not cluster within any defined clade on the phylogenetic tree; instead, strains appeared as standalone lineages. Clade 1 (light pink branch) contains strains from the United States, the United Kingdom, Canada, Germany, and South Africa isolated from different sources. In clade 2 (green branch), a total of 12 *S.* Mbandaka ST413 were grouped, with the majority consisting of 11 strains originating from the United Kingdom, including 91% (10/11) human isolates, and 9% (1/11) isolates from food, and beyond a single strain isolated from food in India (Figure 5). 

Clade 3 (purple branch) was well-defined and comprised three poultry strains isolated in Brazil, specifically, the isolates 1158/18, 1096/18, and 1095/18 from our study, indicating a close phylogenetic relationship between these strains, whereas clade 4 (yellow branch) encompassed isolates predominantly derived from the United Kingdom (95.9%), and a smaller portion from United States strains (4.1%). The *S.* Mbandaka ST413 strains from clade 5 (red branch) all originated from the United Kingdom, with 11 strains isolated from food sources (64.7%) and six from human hosts (35.3%), out of a total of 17 strains. Clade 6 (blue branch) represented the largest assemblage of isolates, encompassing Brazilian genomes of *S.* Mbandaka ST413 1124/18, 1092/18, and 1097/18, which exhibited a closer phylogenetic affinity to other strains isolated from poultry and food sources from the United Kingdom (Figure 5).

## 4. Discussion

Our findings present genomic features of the new six genomes of *S*. Mbandaka ST413 and evolutionary insight into the dynamics of this serovar with sequences from different regions of the world. This sequence type has been implicated in foodborne outbreaks in the EU/EEA, Israel, and the UK [7,41] and identified in cases of salmonellosis in Brazil [42]. The recent identification of *S*. Mbandaka ST413 in clinical cases highlights the importance of understanding its genetic characteristics. However, in Brazil, the lack of epidemiological data on this serovar in both humans and poultry led to an unknown scenario, making it difficult to prevent outbreaks and control strategies.

The phylogenetic tree suggests an epidemiological link in the spread of *S.* Mbandaka ST413, with sequences from Brazilian poultry closely related to those from food and human isolates in the United Kingdom and the United States. Non-typhoid serovar *Salmonella enterica* has been a burden to public health, especially for groups of immunocompromised individuals who can develop serious infections that result in death [43]. Thus, the fact that the sequence of *S.* Mbandaka 1124/18 isolated from poultry in Brazil is closely linked to SALMB7937AA (a human isolate from the United Kingdom) raises an alert for the need for health surveillance since *S.* Mbandaka ST413 was associated with outbreaks in Europe, with cases of septicaemia and one person dying; the most likely the cause of this outbreak was the consumption of contaminated chicken meat [7]. In 2019, a study reported the case of a 69-year-old immunocompromised man who developed mitral valve endocarditis contracted by cereal consumption during an *S*. Mbandaka outbreak in the United States (2018). The authors report the difficulty of treating this infection for this patient due to the MDR feature of that strain [44]. The arrangement of a clade with sequences 1097/18 and 1092/18 with a sister group of food isolates derived from the United Kingdom reinforces the need for control and prevention measures for these pathogens in products of animal origin, which are one of the forms of transmission of infection to humans and are often associated with food outbreaks [7].

Several virulence genes are clustered within SPIs on the chromosome of strains of *S. enterica* and are involved in cell invasion, intracellular survival, and inflammation [45]. The presence of C63PI (six/six) and SPI 9 (six/six) was noticed herein in all *S.* Mbandaka genomes. Although SPIs 1-5 are common in *Salmonella* serovars [46], there are divergences regarding the presence and absence of genes in these islands [45,47]. Many of those mainly found in SPI 2 encode structural and effector proteins of a type III secretion system (T3SS) that transfer essential virulence effectors with important functions during cell infection [45]. Although *Salmonella* Typhi has specific virulence factors, many effectors are absent in this serovar and present in nontyphoidal *Salmonella* (such as *S.* Mbandaka) related to wide-range hosts [48]. Notably, four/six of the *S.* Mbandaka genomes analysed herein lack *sseE*, *sseC*, and *sseD* genes in SPI 2 (Figure 3). They compound the *sse* operon (ABCDEFG), in which the *sseC* and *sseD* genes are enrolled to the expression type III secretion system (T3SS) components, and *sseE* has been linked to the possible role of chaperone or helper function for SPI 2 dependent type III reinforcement [49,50]. This implies that the absence of those key genes in SPI 2 of *S.* Mbandaka can lead to the potential attenuation of virulence; however, this hypothesis needs validation through in vivo experiments, not addressed in this work.

The analysis of *S.* Mbandaka virulence factors in the present study also showed the absence of *sseA*. In vivo experimentation using *S.* Typhimurium with *sseA* mutation has shown that this gene is the chaperone for the translocon components *sseB* and *sseD*, leading to a high level of virulence attenuation and an intracellular replication defect, while a strain with a mutation in *sseE* is still capable of carrying out systemic *Salmonella* infection of the mouse, suggesting that *sseE* is not essential to this virulence system [49,50]. SPI 3 harbours the *mgtCB* virulence genes that are related to Mg^2+^ uptake when under restricted availability of this cation, such as within the macrophage environment [45,51]. While the *mgtCB* genes were predicted in *S.* Mbandaka isolates, other regions of SPI 3 were absent in all the genomes [47], possibly implying that these genes are not essential for *Salmonella* serovar infection. Although the expressions of virulence genes are not the focus of this work, we acknowledge that additional research into the variation in the virulence factors and the interaction of those genes will further our understanding of the role of the virulence mechanism in this serovar and contribute valuable insights into the broader landscape of *Salmonella* pathogenesis.

Additionally, for virulence factors, this study showed that *S.* Mbandaka ST413 strains carry plasmid-borne or chromosomal genes conferring resistance to several antimicrobials commonly used in both the poultry industry and human medicine, such as aminoglycosides (*aac(6′) -Iaa*), sulfonamide (*sul2*), streptomycin (*aph(6)-Id*), tetracycline (*tetA* and *tetB*), and trimethoprim (*dfrA14*) [41]. The presence of the *aac(6′)-laa* gene is in line with the wide distribution of aminoglycoside resistance genes among *Salmonella* strains of different serovars [52,53]. However, despite the prevalence of aminoglycoside resistance genes and their in vitro characteristics, it has been reported that this antimicrobial class exhibits clinical inefficacy against *Salmonella* spp. [54]. 

The plasmid replicon IncHI2A was detected in four *S*. Mbandaka genomes and harboured tetracycline resistance genes (*tet*ACDR). Our findings align with a broader context of antimicrobial resistance in *Salmonella* spp., where an average of 71.1% of tetracycline-resistant isolates and an average of 57.4% of sulphonamide-resistant strains, with the *tetA*, *tetB*, *sul1*, and *sul2* genes being the most prevalent [55]. This underscores the significance of conjugation events in gene dissemination since those are often associated with plasmids [56]. Furthermore, the selective pressure exerted using these antimicrobials in agriculture further fosters the emergence of antibiotic resistance [57].

*bla*_OXA-129_ is an Extended Spectrum β-lactamase (ESBL)-encoding gene that belongs to the class D beta-lactamase enzymes, and it is often carried by plasmids [58]. The presence of pathogens such as *S*. Mbandaka that harbour β-lactamase genes carried by mobile genetic elements is a burden that creates a multifaceted risk, which, allied to the potential inefficiency of antibiotic treatment, facilitates the spread of resistance among several bacterial species [59]. In contrast to previous findings in *Salmonella* spp. related to the poultry industry, the presence of this plasmid-borne gene in our isolates highlights the complexity of the antimicrobial resistance dynamics and the need for continued research on plasmids and other genetic elements that may be involved in its dissemination [60]. 

Another QAC resistance gene (*qacF*) was detected in multidrug-resistant isolates of *S. enterica* from chickens, which was carried in a plasmid pSGB23 along with 11 antimicrobial and disinfectant resistance genes [61]. This corroborates with our study, where all five QAC-resistant sequences are multidrug-resistant strains. The dissemination of these genes can become a problem for public health because QAC is an essential biocide used to disinfect food processing facility environments and to prevent and control nosocomial infections in hospitals [62]. 

In *Salmonella* spp., the main mechanism of resistance to quinolones is associated with mutations in the *gyrA* and *parC* genes [63]. In the present study, all *S.* Mbandaka sequences had a single point mutation in ParC [T57S], a common mutation in *Salmonella* spp. serovars since the early 2000s [64]. *qnr* genes encoding plasmid-mediated resistance to quinolones (PMQR) in *S. enterica* isolated from animals have been reported [65] but were not detected in the *S.* Mbandaka genomes in the present study.

One of the most striking genomic features of the strains studied here is SGI1, which was present in most study genomes (four/six). This resistance island was first identified in a global epidemic of multidrug-resistant *Salmonella* Typhimurium DT104 [66]. A feature of this island is that it often contains various genes endowing their hosts with new traits, like antimicrobial resistance and virulence that enhance bacterial adaptation to the environment [67]. Beyond *Salmonella* serovars, the dissemination of the SGI1 extends to other bacteria that already circulate in humans and production animals (poultry and swine), suggesting that its spread is influenced by various genomic events such as insertions, deletions, and homologous recombination [68,69]. In a study encompassing 35 *Salmonella* serovars, variations in these moving elements were observed, in which the *dfrA12* and *aadA2* genes were the most prevalent [68]. Our study corroborates these findings, with the presence of the *intl* gene in all *S*. Mbandaka genomes accompanied by antimicrobial resistance genes (*sul1*, *aadA2*, and *qacE*), emphasizing the imperative for ongoing surveillance to comprehend and address the spread of genetic elements associated with the determinants of resistance.

While *S*. Mbandaka’s multidrug-resistant profile has been acknowledged, little is understood about the genetic mechanisms behind this trait and the transferability of resistance [29]. Our study identified the IncHI2A plasmid replicon in four *S*. Mbandaka genomes as a key vehicle for horizontally transferring tetracycline (*tet*) and mercury (*mer*) resistance genes. The plasmid also carries genes facilitating transfer mechanisms (*trh* e htd). The IncHI2 plasmid is implicated in spreading antibiotic resistance genes in clinical and food isolates of *Salmonella*, such as β-lactamase (*bla_OXA_*_-1_ and *bla_TEM_*_-1_), and Plasmid-Mediated Quinolone Resistance (*qnrA* and *acc (6′) -ib-cr*), with potential implications for clinical and public health [70].

Due to the extensive diversity within the *Salmonella* genus, identifying phages and interactions with the host cell is crucial, given their role in bacterial virulence, significant potential as biocontrol agents, and for use as therapeutics [71]. The *Enterobacteria* phage ST64T, previously identified in *S*. Typhimurium, integrates near tRNAs and was found to be associated with tRNA-Arg (anticodon CCT) in our study with *S.* Mbandaka, facilitating pathogenicity islands integration, which can affect gene expression and modulate virulence [72]. While temperate phages like SW9 and phiV10 are related to aid *Salmonella* adaptation during infection [73], further investigation is needed on phiV10 integration in the *Salmonella* genus, given its specific association with *E. coli* O157:H7 [74], which is associated with severe intestinal infections [75]. Despite the diversity of phages described in this study, no genes encoding virulence or antibiotic resistance were predicted in the 10 phage sequences; however, our understanding of the ecology and diversity of *S.* Mbandaka phages is limited, and it is imperative to conduct additional tests to confirm the absence of these genes.

## 5. Conclusions

This study showed some *S*. Mbandaka ST413 strains circulating in egg-laying flocks in the Brazilian southeast harbour genomic features associated with strong antimicrobial resistance profiles, which is a hallmark of epidemiologically relevant strains. They also have conserved virulence machinery and are genetically close to strains involved in foodborne outbreaks and invasive salmonellosis cases around the world, granting the need for further research on the factors behind the emergence of this *Salmonella* serovar. This study expands our knowledge of *S*. Mbandaka ST413 and highlights the necessity for further investigations into the genetic and environmental determinants contributing to the evolution and dissemination of these serovars.

## Figures and Tables

**Table 1:** Quality features of draft genome assemblies of the *S.* Mbandaka strains from the caecal content of the laying quail (1092/18) and laying hens (1095/18, 1096/18, 1097/18, 1124/18, and 1158/18) in this study.

Sample ID						
Assembly Attributes	1092/18	1095/18	1096/18	1097/18	1124/18	1158/18
Strain ID	SAMN37152295	SAMN37152292	SAMN37152296	SAMN37152297	SAMN37152298	SAMN37152299
Genome size (bp)	4,854,182	4,929,569	4,929,238	4,738,760	4,934,713	4,916,385
Contigs number	41	36	37	28	51	52
% GC	52.2	51.9	51.9	52.2	51.9	51.8
L_50_	5	6	7	6	7	8
N_50_ (bp)	322,596	296,604	277,541	286,548	187,348	160,426
Average depth of coverage	108	75	45	27	52	36
CDSs	4956	4936	4977	4753	5010	4993
rRNA	11	7	8	9	10	8
tRNA	80	78	77	75	79	76

N_50_: represents the sequence length of the shortest contig at which 50% of the total assembly length is reached; L_50_: represents the minimal count of contigs needed so that their total length equals half of the genome size; CDSs: Coding Sequences; rRNA: Ribosomal Ribonucleic Acid; tRNA: Transfer Ribonucleic Acid.

**Table 2:** Genotypic and phenotypic characterization of the antimicrobial resistance profiles of *S.* Mbandaka isolated from a quail and laying hen farms in Brazil.

Sample ID	AMR Phenotypes *	Resistance Gene							
		*aac(6′)-Iaa*	*aadA1/dfrA21*	*sul1*	*dfrA25*	*tet(A)*	*tet(B)*	*bla_OXA-129_*	*qacE*
1092/18	StrSulSxtTetOxiCip	+	−	+	+	+	−	−	+
1095/18	StrSulSxtTetOxiAmoAmpAmcCip	+	+	+	−	−	+	+	+
1096/18	StrSulSxtTetOxiAmoAmpAmcCip	+	+	+	−	−	+	+	+
1097/18	StrSulCip	+	−	−	−	−	−	−	−
1124/18	StrSulSxtTetOxiAmoAmpAmc	+	+	+	−	−	+	+	+
1158/18	StrSulSxtTetOxiAmoAmpAmc	+	+	+	−	−	+	+	+
Total		6	4	5	1	1	4	4	5
%		100	66.7	83.3	16.7	16.7	66.7	66.7	83.3

*: Previous study of the phenotypic resistance profile of *S.* Mbandaka isolates carried out by Benevides et al., 2020 [13]; Str: streptomycin; Sul: sulfonamide; Sxt: trimethoprim/sulfamethoxazole; Tet: tetracycline; Oxi: oxytetracycline; Cip: ciprofloxacin; Amo: amoxicillin; Amp: ampicillin; Amc: amoxicillin/clavulanic acid.

**Table 3:** General features and accession numbers of the predicted homologous plasmids with PlasmidFinder of *S.* Mbandaka isolated from the caecal content of commercial egg-laying flocks in Brazil.

Sample ID	Incompatibility Group	Size (bp)	ORFs	NCBI Accession	Homologous Plasmid
1095/18, 1096/18, 1097/18, 1124/18, and 1158/18	IncHI2A	274,762	295	BX664015	R478
1092/18	IncN	50,969	64	AY046276	R46

**Table 4:** Genomic features of prophages detected in six *S.* Mbandaka genomes analysed from the caecal content of a commercial laying quail and hens, São Paulo, Brazil.

Genome	Region Length ^a^	Total Proteins	Contig	Region Position ^b^	Phage Identity	Access Number	% GC
1092/18	46.4 Kb	45	11	3–46,484	*Escherichia* phage phiV10	NC_007804	49.99
	26.7 Kb	34	15	66,909–93,641	*Enterobacteria* phage Fels-2	NC_010463	49.10
	24.7 Kb	31	24	2–24,741	*Enterobacteria* phage HK542	NC_019769	53.23
1095/18	36.3 Kb	45	1	399,882–43,6265	*Salmonella* phage SW9	NC_049459	52.82
1096/18	36.4 Kb	45	2	185,676–222,104	*Salmonella* phage SW9	NC_049459	52.83
1097/18	47.7 Kb	54	9	152,566–200,279	*Enterobacteria* phage ST64T	NC_004348	48.57
	46.4 Kb	45	13	3–46484	*Escherichia* phage phiV10	NC_007804	49.99
	38 Kb	46	18	42,660–80,693	*Enterobacteria* phage Fels-2	NC_010463	50.29
1124/18	36.3 Kb	45	2	455,491–491,874	*Salmonella* phage SW9	NC_049459	52.82
1158/18	36.4 Kb	45	1	138,261–174,689	*Salmonella* phage SW9	NC_049459	52.82%

^a^: The length of the sequence of that region; ^b^: The start and end positions of the region. Only intact (score > 90) phage regions were considered [29,30].

## Data Availability

All data needed to evaluate the results and conclusions can be found in online repositories and the associated Supplementary Material. Additional data related to this study can be requested from the authors.

## Supplementary Materials

The following supporting information can be downloaded at: https://www.mdpi.com/article/10.3390/microorganisms12020312/s1, Figure S1: Heatmap comprising the presence (black cells) or absence (white cells) of 67 virulence genes among Brazilian *S.* Mbandaka isolates. The strains are clustered by their prediction of gene similarities. The strains were analysed with hierarchical clustering using Pearson’s correlation coefficient. The virulence gene profiles that exhibited differentiation among the studied genomes were used to generate the heat map. *Salmonella* Typhimurium LT2 (chromosome: NC_003197) was used as the reference genome.; Figure S2: Genomic maps of *S.* Mbandaka prophages using multiple alignments of the genomic sequence [27]. The CDSs are shown in white, and the connected blocks indicate matching regions between the phages. The phage alignment sequence: *Enterobacteria* phage Fels-2 (1092/18 and 1097/18), *Escherichia* phage phiV10 (1092/18 and 1097/18), and *Salmonella* phage SW9 (1095/18, 1096/18, 1124/10, and 1158/18).; Table S1: Metadata for the 475 *S.* Mbandaka genomes retrieved from the Enterobase database to perform phylogenetic analysis.
