# Supplementary figures and images for: Genomic Features and Phylogenetic Analysis of Antimicrobial-Resistant Salmonella Mbandaka ST413 Strains
Source: Microorganisms. 2024 Feb 1;12(2):312. doi: 10.3390/microorganisms12020312 (PMC10893270; doi:10.3390/microorganisms12020312)

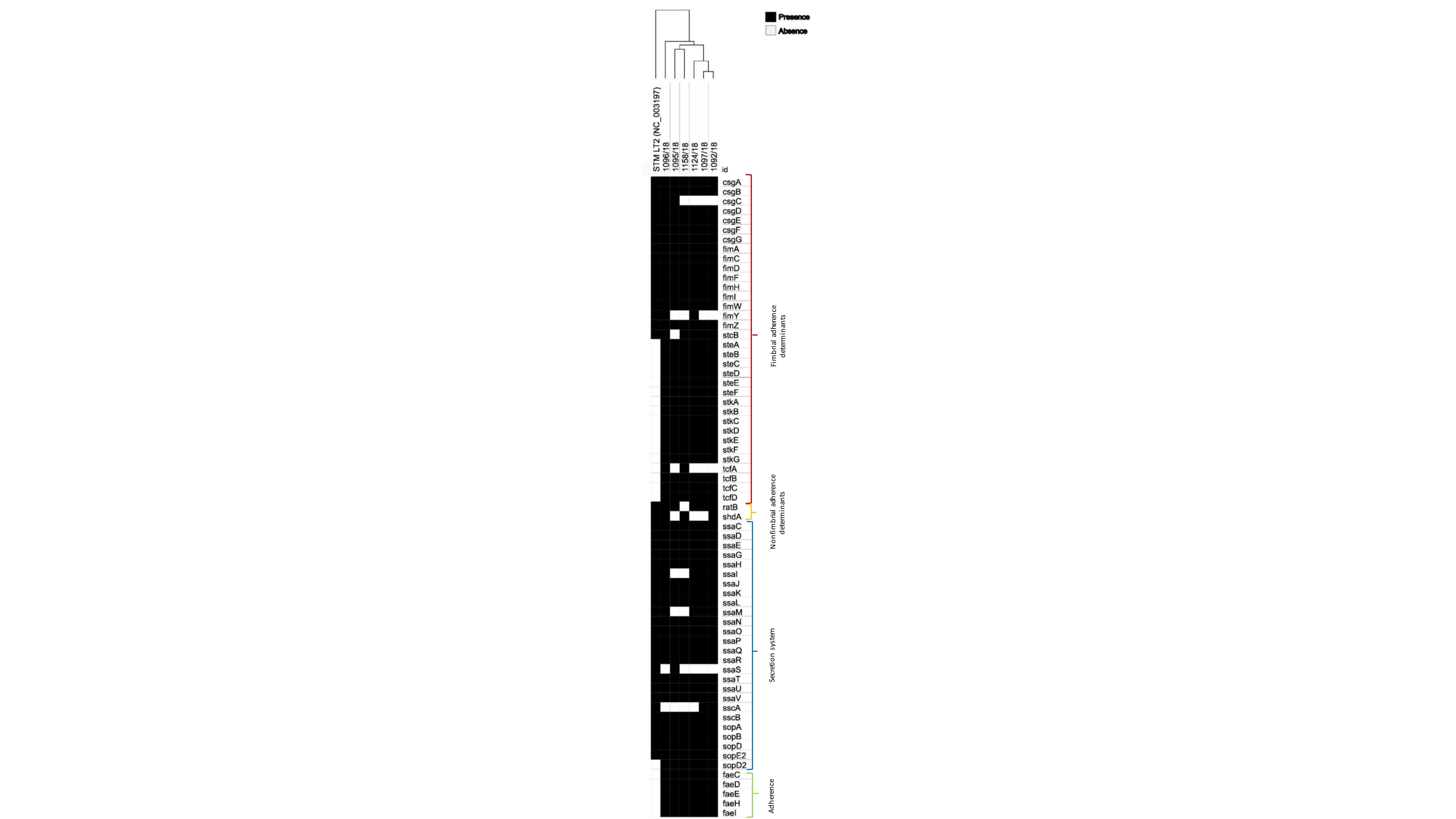

Supplement: Supplementary file 1 [file microorganisms-12-00312-s001.zip › Figure_S1.jpg]
